# Properties Investigation and Damage Analysis of GaN Photoconductive Semiconductor Switch Based on SiC Substrate

**DOI:** 10.3390/mi15101178

**Published:** 2024-09-24

**Authors:** Jiankai Xu, Lijuan Jiang, Ping Cai, Chun Feng, Hongling Xiao, Xiaoliang Wang

**Affiliations:** 1 Laboratory of Solid State Optoelectronics Information Technology, Institute of Semiconductors, Chinese Academy of Sciences, Beijing 100083, China; jkxu@semi.ac.cn (J.X.); caiping@semi.ac.cn (P.C.); cfeng@semi.ac.cn (C.F.); hlxiao@semi.ac.cn (H.X.); xlwang@semi.ac.cn (X.W.); 2 Center of Materials Science and Optoelectronics Engineering, University of Chinese Academy of Sciences, Beijing 100049, China; 3 Beijing Key Laboratory of Low Dimensional Semiconductor Materials and Devices, Beijing 100083, China

**Keywords:** photoconductive semiconductor switch (PCSS), AlGaN/GaN, high power, breakdown

## Abstract

The GaN photoconductive semiconductor switches (PCSSs) with low leakage current and large on-state current are suitable for several applications, including fast switching and high-power electromagnetic pulse equipment. This paper demonstrates a high-power GaN lateral PCSS device. An output peak current of 142.2 A is reached with an input voltage of 10.28 kV when the GaN lateral PCSS is intrinsically triggered. In addition, the method of retaining the AlGaN/GaN heterostructure between electrodes on PCSSs is proposed, which results in increasing the output peak current of the PCSS. The damage mechanism of the PCSS caused by a high electric field and high excitation laser energy is analyzed. The obtained results show that the high heat generated by the large current leads to the decomposition of GaN, and thus, the Ga forms a metal conductive path, resulting in the failure of the device.

## 1. Introduction

Photoconductive semiconductor switches (PCSSs) have unique characteristics, such as high switching speeds, low jitter and high power, which are suitable for several application domains, including fast switching and high-power electromagnetic pulse equipment [1]. Its working principle consists of irradiating laser light on semi-insulating semiconductor materials to generate photogenerated carriers, which significantly decreases the resistivity between the electrodes and performs the conduction of the device [2]. In response to the demand for high off-resistance and low on-resistance switching devices, the semiconductor materials selected for the PCSS have transitioned from GaAs and Si materials [3,4,5,6] to wide bandgap materials, such as SiC [7,8,9] and GaN [10,11,12,13,14,15,16,17]. The GaN material has high critical breakdown field strength, a large band gap, and high electron drift velocity [18,19,20,21,22]. In addition, semi-insulating high-resistance GaN materials can be constructed by introducing Fe impurities [10,11,23]. Due to the aforementioned advantages, GaN-based PCSS devices have received significant attention from researchers. However, some problems should be urgently solved. One consists of implementing an ohmic contact electrode with lower resistivity on the semi-insulating GaN material, which can significantly decrease the resistance of the device when it is turned on. In addition, for PCSS devices operating under high electric fields and large currents, significant thermal effects and the breakdown of the device can result in its state degradation and even its damage [24,25,26].

In this study, AlGaN/GaN structural materials are used on SiC substrates. A high concentration of two-dimensional electron gas (2DEG) can be generated in the potential well on the GaN side of the AlGaN/GaN interface through spontaneous and piezoelectric polarizations. By depositing metal on top of the AlGaN/GaN structural material and annealing it, good ohmic contact can be achieved, and the electrode can be connected to the 2DEG below. The semiconductor material in the area between the metal electrodes is etched, allowing the semi-insulating high-resistance GaN to be left underneath to achieve higher resistance in the off state. GaN photoconductive switching devices with higher power can be obtained by designing different electrode gaps and material structures between electrodes. In addition, the device damage is analyzed, which provides an important basis for the subsequent studies.

## 2. Experiments

**Figure 1:**
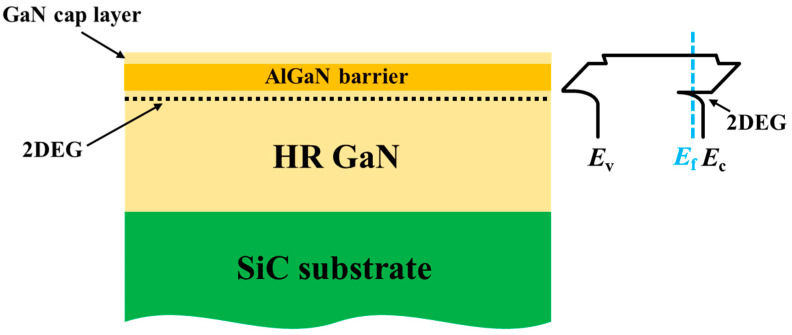
Schematic of the structure of as-grown samples and its band-edge energies.

In this study, the samples were grown on 4-inch SiC substrates through self-made metal–organic chemical vapor deposition (MOCVD). Figure 1 shows the schematic of the AlGaN/GaN heterostructure and its band-edge energies. Acting as a deep acceptor, iron is introduced into the GaN to compensate for inherent n-type conductivity, resulting in a high-resistivity GaN for high off-state bias voltage. For the AlGaN barrier layer with a GaN cap having a thickness of 3 nm, the Al content and thickness measured by the PANalytical X’Pert PRO MRD (Malvern Panalytical, Almelo, The Netherlands) were equal to 23% and 22 nm, respectively. The density of the 2DEGs and mobility of the heterostructure, measured by the non-contact Hall (LEI 1600), were equal to 9.19 × 10^12^ cm^−2^ and 2014 cm^2^/V·s, respectively.

**Figure 2:**
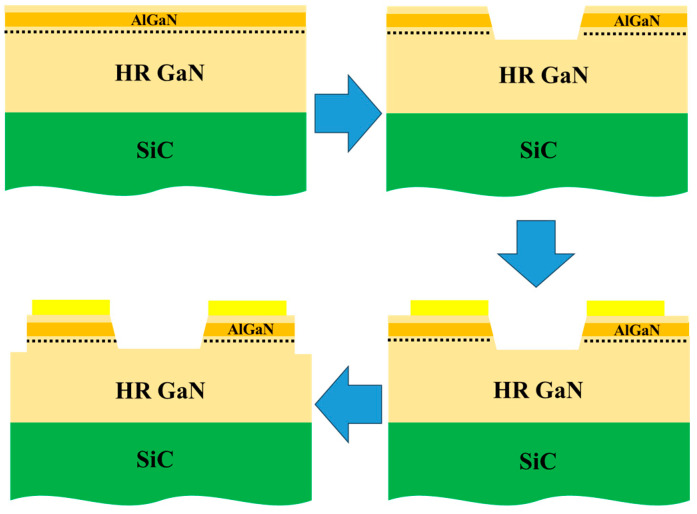
The fabrication process for the GaN PCSS.

The devices were fabricated using the processes shown in Figure 2. A trench of 460 nm between two electrodes was first achieved by inductively coupled plasma etching (ICP) due to the restrained impact of the trench on the high local electric field. Ti/Al/Ni/Au was then deposited by electron-beam evaporation to produce the electrodes. Annealing at 870 °C for 30 s in N_2_ ambient was performed to form the ohmic contacts. Metal-nitride clusters—such as TiN—that penetrate through the AlGaN barrier layer are the direct link between the 2DEG and the metal [27,28,29]. Using the transmission line model, the measured specific contact resistance was equal to 7.2 × 10^−4^ Ω·cm^2^. Afterward, the mesa isolation, having a depth of 460 nm, was achieved by ICP.

**Figure 3:**
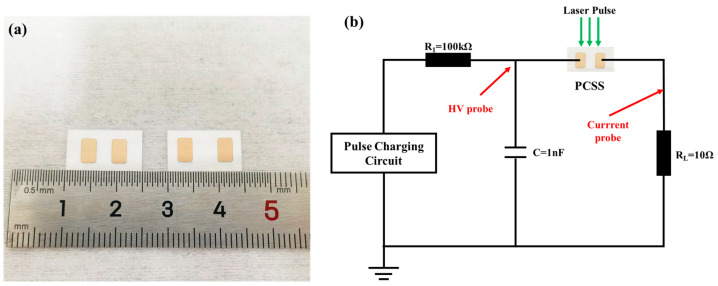
(**a**) Image of the PCSS with electrode gaps of 3 mm and 5 mm and the (**b**) principle circuit for testing.

The devices with electrode gaps of 3 mm and 5 mm, shown in Figure 3a, were used. The device exhibiting a larger electrode gap has a higher resistance, which results in a smaller current in the circuit when the PCSS is turned on. The testing circuit is shown in Figure 3b. It consists of a 1 nF capacitor and a load resistor assembly (*R_L_*) of 10 Ω. The pulse-charging circuit charges the capacitor up to operation voltage, which is measured by the HV probe. When a laser pulse is incident on the switch, the latter turns on, discharging the capacitor through the load resistance. The capacitor behaves like a voltage source to supply the PCSS. The load current is measured by a current probe, and the optical energy is also monitored by an optical waveform detector.

The maximum output power (*P_out_*) on the load (*R_L_*) and the maximum power of the photoconductive switch (*P_pcss_*) are expressed as:

(1) $$P_{out}=I_{peak}^{2}R_{L}$$

(2) $$P_{pcss}=(U_{in}-I_{peak}R_{L})I_{peak}$$

## 3. Results and Discussion

**Figure 4:**
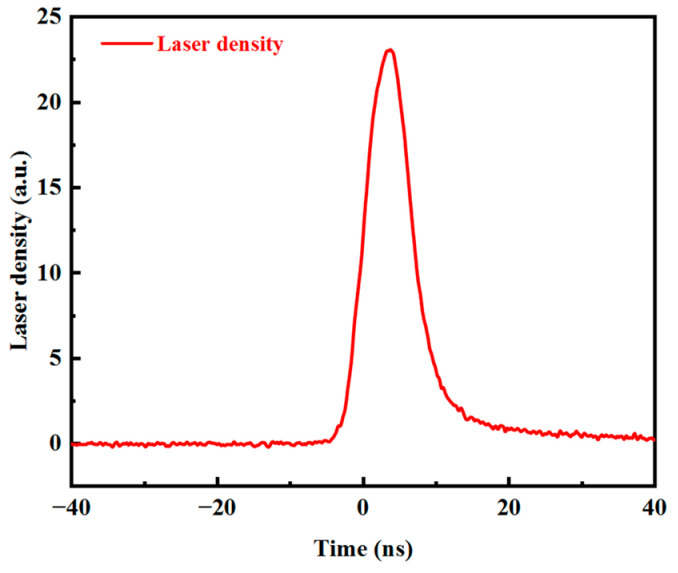
Laser waveform of 355 nm wavelength.

**Figure 5:**
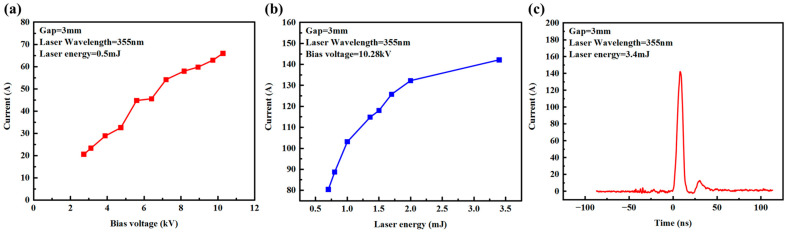
(**a**) Current response with different bias voltages, (**b**) variations in the peak current with the laser energy, and (**c**) photocurrent waveform with laser energy of 3.4 mJ.

The laser wavelength of 355 nm, obtained after frequency tripling of the Q-switched Nd: YAG laser, is used as the trigger source. The pulse frequency of the laser is 1 Hz, which is consistent with that of the input voltage applied across the capacitor. Figure 4 shows the waveform of the laser, whose full width at half maxima (FWHM) is equal to 7.4 ns. Figure 5a shows the peak photocurrent function of the input bias voltage, which varies between 0 kV and 10 kV for an optical energy of 0.5 mJ. Since the laser energy is fixed, the concentration of the photogenerated carriers involved in conduction remains unchanged. At this time, the directional movement of the photogenerated carriers under the external electric field forms a current, which increases with the increase in external voltage. It can be seen from Figure 5b that when the applied voltage is set to 10.28 kV, as the laser energy increases, the photocurrent also increases and tends to saturate. This is mainly due to the fact that when the concentration of the photogenerated carriers increases with the increase in incident laser energy, the impact of the resistance of the PCSS on the total resistance decreases, and thus, the current approaches saturation. Figure 5c shows the photocurrent waveform when the laser energy reaches 3.4 mJ. The photocurrent peak reaches 142.2 A, and the output power reaches 202.2 kW. However, when the laser energy continues to increase, the device exhibits a breakdown, and cracks appear on its surface.

**Figure 6:**
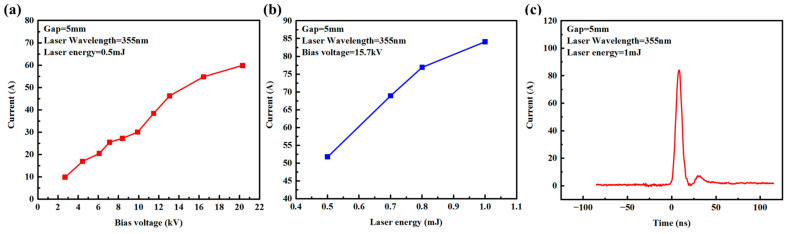
(**a**) Current response with different bias voltages, (**b**) variations in the peak current function of the laser energy, and (**c**) photocurrent waveform with laser energy of 1 mJ.

Since the 5 mm gap device has a larger electrode gap, it is expected to achieve a higher withstand voltage. In fact, the applied voltage is increased to 20 kV and the device can still normally operate. Device conduction testing was performed for the PCSS with an electrode gap of 5 mm. It can be seen from Figure 6a that when the laser energy is fixed at 0.5 mJ, for the PCSS with an electrode gap of 5 mm, the peak current increases with the increase in the applied voltage, and the maximum voltage can reach 20.32 kV. It can be observed from Figure 6b that when the applied voltage is fixed at 15.7 kV, the peak photocurrent increases with the increase in laser energy. When the laser energy increases to 1 mJ, the peak photocurrent reaches 84.1 A. Its waveform is shown in Figure 6c.

**Figure 7:**
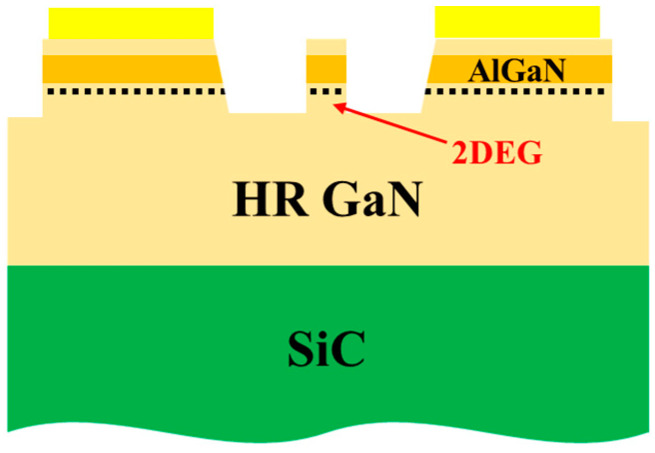
Schematic diagram of the structure with 1/5 retaining AlGaN/GaN heterostructure between electrodes.

The device exhibiting a larger electrode gap has a higher resistance, which results in a smaller current in the circuit when the PCSS is turned on. Therefore, a 1/5 gap length of the AlGaN/GaN structure material is retained based on the device having an electrode gap of 5 mm, and its 2DEG can be used as a low resistive area that participates in conducting electricity. A schematic diagram of the structure is shown in Figure 7.

**Figure 8:**
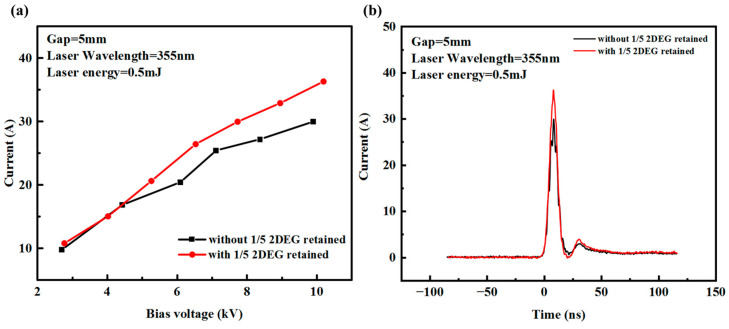
(**a**) Current response with different bias voltages for two structures and (**b**) photocurrent waveform of two structures with 10 kV bias voltage.

The characteristics of the 5 mm gap PCSS with or without the 2DEG part were also compared. The obtained results showed that the device with a 1/5 gap length of the AlGaN/GaN structure material has a higher peak photocurrent for the same laser energy and applied voltage, as shown in Figure 8a. Figure 8b shows the photocurrent waveforms of the two structures for a laser energy of 0.5 mJ and an applied voltage of 10 kV. This is because the low-resistance area formed by the 2DEG allows the PCSS to have smaller resistance when it is turned on.

**Figure 9:**
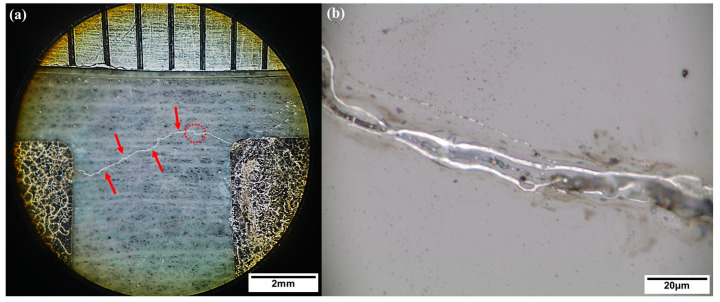
Microscope images of the PCSS after breakdown: (**a**) the breakdown path (marked by the red arrows) between electrodes and (**b**) an enlarged view of the area surrounded by the dashed line.

**Figure 10:**
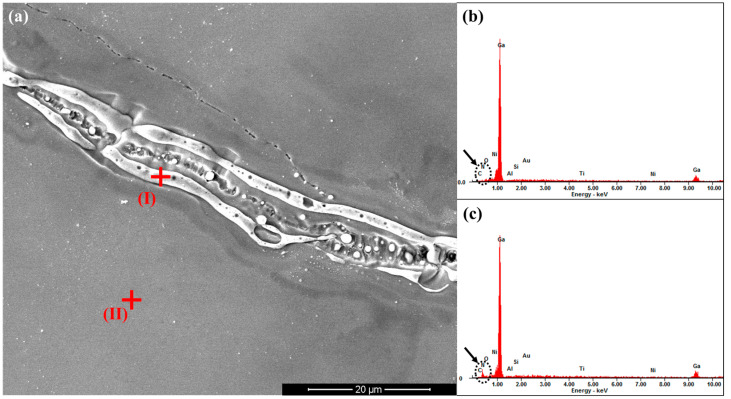
(**a**) SEM scan of the breakdown path and EDS energy spectra of the selected measurement point (**b**) (I) and (**c**) (II) marked by red crosses.

The PCSS device after the breakdown was also studied, and the cause of the failure was analyzed. It can be seen from Figure 9a that a breakdown path between two electrodes appeared after the device failed. Note that the location of the breakdown path is marked by the red arrows. Figure 9b presents the enlarged view of the area marked by the dashed line in Figure 9a. It can be seen from the enlarged image that in the breakdown path, in addition to the GaN materials, bright substances also exist. To determine the composition of this substance, scanning electron microscope energy dispersive system (SEM-EDS) measurements were performed on the sample. The SEM scan is shown in Figure 10a, where the red crosses are the two selected measurement points: (I) the bright position in the breakdown path and (II) the GaN surface outside the breakdown path. Figure 10b,c shows the EDS energy spectra of (I) and (II), respectively. It can be seen that in the bright position, the Ga element is the elementary substance, and the peak of the N element hardly appears. The peak of the N element can be seen in the GaN area outside the channel. The atomic percentage results for the two positions are shown in Table 1. Through quantitative detection, this study believes that the bright substance in the breakdown path should be gallium. It can be assumed that when high voltage and high laser energy are applied to the PCSS device, defects and damage on the surface of the GaN material will form a leakage path, such as nitrogen vacancy-related shallow donors in the near-surface introduced by ICP etching [30]. The current density in the leakage path is very high, which results in a lot of heat. Therefore, when the temperature is high enough to decompose GaN, the N element escapes from the surface, and the Ga element is left in the leakage path, connecting the two electrodes together. The causes and modes of the breakdown damage of the GaN PCSS devices are very different from those of the SiC PCSS [25,26]. This issue will be tackled in future work.

## 4. Conclusions

In this study, high-power GaN PCSS devices are fabricated on a SiC substrate using GaN-based epitaxial materials. For a PCSS with a 3 mm electrode gap, a 355 nm wavelength laser having an energy of 3.4 mJ is used for intrinsic triggering. When the applied voltage is 10.28 kV, the output peak current reaches 142.2 A, and the output power reaches 202.2 kW, which is at a high level. In order to withstand higher voltages (the maximum withstand voltage can reach 20 kV), a PCSS with a gap of 5 mm was used, but it has a smaller photocurrent. In order to achieve a lower on-resistance of PCSS devices and reduce energy consumption, it is proposed to retain the AlGaN/GaN heterostructure between electrodes on the PCSS. The characteristics of the 5 mm gap PCSSs with or without the 2DEG part are compared. The obtained results show that the device with a 1/5 gap length of 2DEG has a higher peak photocurrent. This is due to the fact that the low-resistance area formed by the 2DEG allows the PCSS to have smaller resistance when it is turned on, which provides a new idea for the low-on-resistance design of GaN PCSS devices. The damage mechanism of the GaN PCSS is analyzed. The breakdown of GaN PCSS devices is mainly caused by the high heat generated by large currents. The heat makes N escape from the material, and Ga forms a metal conductive path, which results in device failure. This study provides a foundation for the optimization of the reliability of devices.

## Figures and Tables

**Table 1:** The atomic percentage results for the two positions.

Elements	C	N	O	Ga	Al	Si	Au	Ti	Ni
Bright position	4.72%	0.00%	5.02%	86.45%	0.00%	0.58%	0.82%	1.16%	1.25%
GaN	0.00%	35.69%	1.51%	57.19%	1.43%	1.43%	0.51%	1.25%	0.97%

## Data Availability

The original contributions presented in the study are included in the article, further inquiries can be directed to the corresponding author.

## References

[1] Sullivan J.S., Stanley J.R. (2008). Wide Bandgap Extrinsic Photoconductive Switches. IEEE Trans. Plasma Sci..

[2] Mazzola M.S., Schoenbach K.H., Lakdawala V.K., Germer R. (1989). GaAs photoconductive closing switches with high dark resistance and microsecond conductivity decay. Appl. Phys. Lett..

[3] Zutavern F.J., Loubriel G.M., Helgeson W.D., O’Malley M.W., Gallegos R.R., Hjalmarson H.P., Baca A.G., Plut T.A. (1994). High-gain GaAs photoconductive semiconductor switches PCSS device lifetime high-current testing optical pulse generators. Optically Activated Switching IV.

[4] Wu T., Shang B., Vorst A.V. (1996). A high speed silicon photoconductive switch. Microw. Opt. Technol. Lett..

[5] Liu T.A., Lin G.R., Lee Y.C., Wang S.C., Tani M., Wu H.H., Pan C.L. (2005). Dark current and trailing-edge suppression in ultrafast photoconductive switches and terahertz spiral antennas fabricated on multienergy arsenic-ion-implanted GaAs. J. Appl. Phys..

[6] Liu Y., Shen Y., Wang W., Xia L., Ye M., Zhang H., Shi J., Deng J. Solid-state pulsed power system with GaAs-PCSS for DWA. Proceedings of the IEEE 21st International Conference on Pulsed Power.

[7] Wu Q., Xun T., Zhao Y., Yang H., Huang W. (2019). The test of a high-power semi-insulating linear-mode vertical 6H-SiC PCSS. IEEE Trans. Electron Dev..

[8] Wu Q., Zhao Y., Xun T., Yang H., Huang W. (2019). Initial test of optoelectronic high power microwave generation from 6H-SiC photoconductive switch. IEEE Electron Dev. Lett..

[9] Chu X., Meng J., Wang H., Zhu D., Yuan Y., Huang L., Xiang Z., Han J., Deng B., Cui Y. (2024). Influence of Nitrogen Doping in Vanadium- Compensated 4H-SiC on Transient Photocurrent Response for Photoconductive Microwave Generation. J. IEEE Photonics Technol. Lett..

[10] Wang X., Mazumder S.K., Shi W. (2015). A GaN Based Insulated Gate Photoconductive Semiconductor Switch for Ultrashort High-Power Electric Pulses. IEEE Electron Dev. Lett..

[11] Chen Y., Lu H., Chen D., Ren F., Zhang R., Zheng Y. (2016). High voltage photoconductive semiconductor switches fabricated on semi-insulating HVPE GaN Fe template. Phys. Status Solidi C.

[12] Shi D., Jiang L., Wang Q., Feng C., Xiao H., Li W., Wang X. (2021). A novel structure to enable low local electric field and high on-state current in GaN photoconductive semiconductor switches. Opti. Commun..

[13] Koehler A.D., Anderson T.J., Khachatrian A., Nath A., Tadjer M.J., Buchner S.P., Hobart K.D., Kub F.J. (2017). High voltage GaN lateral photoconductive semiconductor switches. J. Solid State Sci. Technol..

[14] Leach J.H., Metzger R., Preble E.A., Evans K.R. High voltage bulk GaN-based photoconductive switches for pulsed power applications. Proceedings of the Gallium Nitride Materials and Devices VIII.

[15] Meyers V., Mauch D., Kuryatkov V., Nikishin S., Dickens J., Neuber A., Ness R. Toward the development of an efficient bulk semi-insulating GaN photoconductive switch. Proceedings of the IEEE 21st International Conference on Pulsed Power.

[16] Hu L., Huang J., Yang X., Shen X., Sun Y. (2023). Analysis of the Avalanche Operation of a GaN Photoconductive Semiconductor Switch. IEEE T. Electron Dev..

[17] Cai P., Jiang L., Xu J., Xiao H., Feng C., Wang Q., He T., Zhou M., Wang X. (2024). Design of a lateral photoconductive semiconductor switch with a low resistivity region on semi-insulating GaN to enhance breakdown characteristics. Opti. Commun..

[18] Haziq M., Falina S., Manaf A.A., Kawarada H., Syamsul M. (2022). Challenges and Opportunities for High-Power and High-Frequency AlGaN/GaN High-Electron-Mobility Transistor (HEMT) Applications: A Review. Micromachines.

[19] Liu A.-C., Tu P.-T., Langpoklakpam C., Huang Y.-W., Chang Y.-T., Tzou A.-J., Hsu L.-H., Lin C.-H., Kuo H.-C., Chang E.Y. (2021). The Evolution of Manufacturing Technology for GaN Electronic Devices. Micromachines.

[20] Chu J., Wang Q., Feng C., Jiang L., Li W., Liu H., Wang Q., Xiao H., Wang X. (2021). Abnormal increase of 2DEG density in AlGaN/GaN HEMT grown on free-standing GaN substrate. Jpn. J. Appl. Phys..

[21] Niu D., Wang Q., Li W., Chen C., Xu J., Jiang L., Feng C., Xiao H., Wang Q., Xu X. (2021). The Influence of the Different Repair Methods on the Electrical Properties of the Normally off p-GaN HEMT. Micromachines.

[22] Rafin S.M.S.H., Ahmed R., Haque M.A., Hossain M.K., Haque M.A., Mohammed O.A. (2023). Power Electronics Revolutionized: A Comprehensive Analysis of Emerging Wide and Ultrawide Bandgap Devices. Micromachines.

[23] Cui L., Yin H., Jiang L., Wang Q., Feng C., Xiao H., Wang C., Gong J., Zhang B., Li B. (2015). The influence of Fe doping on the surface topography of GaN epitaxial material. J. Semicond..

[24] Yang X., Hu L., Yang Y., Huang J., Li X., Liu W., Han C. (2023). Improved Photocurrent for Gallium Nitride Photoconductive Semiconductor Switch by SiO2 Anti-reflection and (SiO2_Ta2O5)6 High-reflection Dielectric Films. IEEE Electron Devi. Lett..

[25] Xiao L., Yang X., Duan P., Xu H., Chen X., Hu X., Peng F., Xu X. (2018). Effect of electron avalanche breakdown on a high-purity semi-insulating 4H-SiC photoconductive semiconductor switch under intrinsic absorption. Appl. Opt..

[26] Zeng L., Wang L., Niu X., Liu F., He T., Gu Y., Yi M., Yao J., Xun T., Yang H. (2024). Characteristics Comparison of SiC and GaN Extrinsic Vertical Photoconductive Switches. IEEE J. Electron Devi..

[27] Wang L., Mohammed F.M., Adesida I. (2008). Formation mechanism of Ohmic contacts on AlGaN-GaN heterostructure electrical and microstructural characterizations. J. Appl. Phys..

[28] Gerbedoen J.C., Soltani A., Mattalah M., Telia A., Troadec T., Abdallah B., Gautron E., De Jaeger J.C. Study of ohmic contact formation on AlGaN-GaN HEMT with AlN spacer on silicon substrate. Proceedings of the European Microwave Integrated Circuits Conference.

[29] Fontserè A., Pérez-Tomás A., Placidi M., Llobet J., Baron N., Chenot S., Cordier Y., Moreno J.C., Gammon P.M., Jennings M.R. (2011). Micro and nano analysis of 0.2 X mm Ti/Al/Ni/Au ohmic contact. Appl. Phys. Lett..

[30] Cao X.A., Zhang A.P., Dang G.T., Ren F., Pearton S.J., Shul R.J., Zhang L. (2000). Schottky diode measurements of dry etch damage in n- and p-type GaN. J. Vac. Sci. Technol. A.

